# Sit-and-Reach Pose Detection Based on Self-Train Method and Ghost-ST-GCN

**DOI:** 10.3390/s25185624

**Published:** 2025-09-09

**Authors:** Shuheng Jiang, Haihua Cui, Liyuan Jin

**Affiliations:** 1 Nanjing University of Aeronautics and Astronautics, Nanjing 210016, China; 2 University of California San Diego, La Jolla, CA 92093, USA

**Keywords:** sit and reach, body keypoints, self-train, pose detection

## Abstract

The sit-and-reach test is a common stretching exercise suitable for adolescents, aimed at improving joint flexibility and somatic neural control, and has become a mandatory item in China’s student physical fitness assessments. However, many students tend to perform incorrect postures during their practice, which may lead to sports injuries such as muscle strains if sustained over time. To address this issue, this paper proposes a Ghost-ST-GCN model for judging the correctness of the sit-and-reach pose. The model first requires detecting seven body keypoints. Leveraging a publicly available labeled keypoint dataset and unlabeled sit-and-reach videos, these keypoints are acquired through the proposed self-train method using the BlazePose network. Subsequently, the keypoints are fed into the Ghost-ST-GCN model, which consists of nine stacked GCN-TCN blocks. Critically, each GCN-TCN layer is embedded with a ghost layer to enhance efficiency. Finally, a classification layer determines the movement’s correctness. Experimental results demonstrate that the self-train method significantly improves the annotation accuracy of the seven keypoints; the integration of ghost layers streamlines the overall detection model; and the system achieves an action detection accuracy of 85.20% for the sit-and-reach exercise, with a response latency of less than 1 s. This approach is highly suitable for guiding adolescents to standardize their movements during independent sit-and-reach practice.

## 1. Introduction

In recent years, the deep integration of computer vision and deep learning technologies has been reshaping research paradigms in sports science. Human pose estimation-based motion detection techniques, combined with temporal analysis and biomechanical modeling, not only enable precise quantification of athletic postures, but also facilitate the development of intelligent “perception-analysis-feedback” closed-loop systems. These advancements provide technical support for injury prevention, training efficiency enhancement, and personalized guidance [1]. In China, the National Student Physical Fitness Standard includes a flexibility test called the sit-and-reach test for university students. This test quantitatively evaluates the range of motion of the trunk, waist, and hip joints in a static state, reflecting the flexibility and muscular fitness of these areas [2]. The exercise requires no expensive equipment—only a flat, clean indoor or outdoor space—and can improve sitting posture, alleviate lower-back discomfort, and enhance overall flexibility, thereby reducing healthcare costs.

Although numerous online tutorials demonstrate the sit-and-reach exercise, adolescents often perform it incorrectly, risking injuries such as muscle strains or joint sprains, which compromise health and training efficacy [3]. Persistent poor posture may also diminish motivation, increase chronic injury risks, and negatively impact test performance or daily life. Thus, ensuring correct form is critical. However, professional coaching is impractical for non-athletes due to cost, while parents lack expertise to assess posture accuracy. With the growing demand for mobile fitness solutions, integrating smartphone cameras and Android-compatible AI applications offers significant advantages. Mobile devices can now run lightweight deep learning models in real time for instant video analysis. AI-based video processing ensures high precision and objectivity, capturing subtle motion differences and eliminating subjective bias. Large-scale motion data analysis enables comprehensive training support. Multimodal fusion (e.g., combining voice and natural language understanding) can further enrich analytical dimensions.

**Figure 1:**
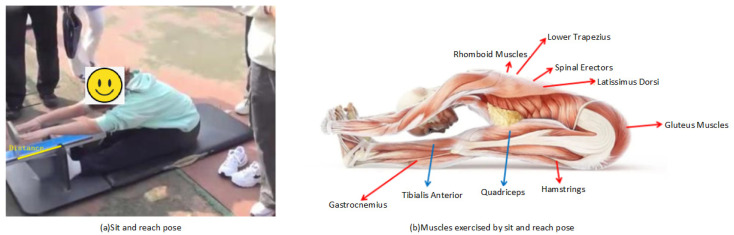
The pose and exercised muscles of the sit-and-reach test.

Current sit-and-reach testers (Figure 1a) fall into two categories: manual scoring by professionals, and sensor-based distance measurement, shown as the yellow line in Figure 1a. However, neither assesses posture correctness, and both are inconvenient for outdoor use due to portability and durability issues. Proper sit-and-reach execution depends on skeletal joint alignment (Figure 1b). (i) Spine: The natural curvature should be maintained, especially for flexible lumbar/thoracic vertebrae for forward flexion. (ii) Hip joints: Crucial flexibility is needed to maximize forward reach. (iii) Knees: These should be fully extended to avoid limiting depth. (iv) Ankles/Shoulders: These should be relaxed to minimize resistance. (v) Arms/Fingers: These should be extended forward to increase reach distance.

Although video-based detection cannot precisely localize muscle tissue, human body keypoints can be used to infer skeletal and joint positions, thereby indirectly estimating muscle state. This enables temporal action analysis. In reference [4], human body keypoints are treated as a graph structure and grouped based on the local connectivity of joints and their limited range of motion. In specific movements, grouped keypoints exhibit coordinated and synchronized changes. This grouping strategy reduces graph complexity and enhances the robustness of feature extraction.

The score in the seated forward bend test is determined by the distance the fingertips extend beyond the soles of the feet. During this test/movement, adolescents often exhibit the following issues when their fingertips reach the farthest point: (1) knee flexion occurs, resulting in an invalid test score; (2) the wrists are not fully extended, leading to suboptimal performance; and (3) insufficient lumbar flexion occurs, also leading to suboptimal performance. Furthermore, two or even all three of these issues may occur simultaneously. This paper categorizes all these issues as erroneous movements and proposes a spatio-temporal graph convolutional network embedded with ghost layers (Ghost-STGCN) for their detection. The primary steps include human body keypoint extraction, keypoint grouping, and Ghost-ST-GCN-based detection.

To address these challenges, this paper proposes the following key contributions, which constitute our primary technical innovations:

(1) This paper proposes an adaptive self-train approach to improve the accuracy of keypoints. It constitutes a fundamental step in sit-and-reach pose detection for keypoint extraction. The self-train method effectively utilizes annotation information from labeled datasets and correlations between unlabeled sit-and-reach video frames for loop training.
(2) It proposes a body keypoint grouping strategy that divides body keypoints into three groups (upper limbs, torso, and lower limbs) according to the characteristics of the sit-and-reach movement, then extracts spatiotemporal graph features in parallel before classifying whether the movement is correct.
(3) It improved the classical ST-GCN structure by embedding ghost modules to its temporal convolutional network (TCN), which ensures detection accuracy while achieving good performance.

## 2. Related Research

The 1948 London Olympics marked the first use of high-speed photography to assist in refereeing sprint events, initiating the application of this technology for sports monitoring and analysis [5]. For instance, in sprint races, judges traditionally relied on visual observation to determine finishing orders, which often sparked controversies in closely contested competitions. High-speed cameras were employed to capture the exact moment of crossing the finish line, enabling manual frame-by-frame analysis to accurately determine athletes’ arrival sequences and resolve outcomes that were difficult to judge with the naked eye. Beyond result determination, high-speed photography has been utilized for athlete movement analysis. In sports such as track and field, swimming, and gymnastics, coaches analyze athletes’ motions frame by frame to identify technical flaws and propose improvements. This technology provides detailed evidence for training optimization. However, reliance on manual analysis presents issues such as subjectivity, inefficiency, and limited precision. With advancements in computer vision technology, its automation, high accuracy, and big data processing capabilities have made it the mainstream solution for sports monitoring [6]. It not only enhances competition fairness and accuracy but also offers scientific support for athlete training and tactical optimization. In particular, implementing body keypoint detection for motion analysis and error correction in sports via mobile devices has become a key research and application focus [7].

In the field of motion detection, the OpenPose model serves as a representative example, which employs deep learning algorithms to identify and track body keypoints (e.g., joints and limb extremities), thereby achieving precise capture of complex movements. In basketball, for instance, Z. Cao et al. utilized the OpenPose model to analyze players’ shooting motions [8]. By comparing discrepancies between the ideal motion model and actual performance, the system guided athletes to adjust their techniques, consequently improving shooting accuracy.

Y. Zhang applied convolutional neural networks (CNNs) to analyze swimmers’ strokes [9], capturing body keypoint positions in water to evaluate movement efficiency and force distribution. This approach not only enhanced the scientific rigor of training but also effectively reduced sports-related injuries.

In soccer training, D. H. Jung implemented computer vision technology to analyze players’ running trajectories and technical movements [10]. Real-time monitoring of positioning and actions provided coaches with data-driven insights for tactical adjustments, significantly improving the team’s overall performance.

T. Yuan explored the application of computer vision technology for video-based motion analysis of track and field athletes [11]. Multiple intelligent methods were integrated to achieve environmental control, rapid video feedback, quantitative physical monitoring, and intelligent technical analysis, as well as fatigue assessment and rehabilitation.

A. Ghosh et al. proposed a novel video analysis framework [12] designed to evaluate and enhance badminton players’ stroke techniques. By analyzing players’ hitting motions and shuttlecock trajectories, the framework provided detailed feedback for technical refinement. The results demonstrated that this method effectively improved players’ hitting accuracy and competitive performance.

R. P. Srivastava presented an application of computer 3D vision technology in yoga training [13]. The researchers developed a real-time monitoring system that analyzed practitioners’ postures and breathing patterns, offering immediate feedback and guidance. This system not only helped practitioners master yoga poses more accurately, but also enhanced training efficacy and safety.

The aforementioned cases demonstrate that intelligent sport motion detection fundamentally relies on spatial and temporal feature extraction and classification. Among these, video-based body keypoint detection serves as the core algorithmic component, which has spurred extensive research efforts. Notably, when combined with mobile-oriented lightweight optimization, these techniques demonstrate enhanced market applicability.

K. Sun proposed HRNet (High-Resolution Network) [14], which maintains high-resolution feature maps through parallel connections between high-to-low resolution subnetworks. This architecture significantly improves body keypoint localization accuracy, particularly excelling in single-person pose estimation tasks. It has achieved state-of-the-art performance on benchmark datasets including COCO and MPII.

X. Zhang developed an enhanced version of ShuffleNet [15] that incorporates lightweight convolutions with channel shuffle operations. This modification substantially reduces computational complexity while maintaining detection accuracy, making it particularly suitable for real-time body keypoint detection on mobile devices.

Y. Chen introduced the Cascaded Pyramid Network (CPN) [16], which progressively refines human keypoint detection results through a cascaded pyramid structure. This approach effectively addresses challenges in multi-person scenarios, particularly occlusion and complex backgrounds, demonstrating superior performance on the COCO dataset.

C. Zheng pioneered PoseFormer [17], representing the first application of Transformer architecture to human pose estimation. By leveraging self-attention mechanisms to capture global contextual information, this model achieves remarkable performance in complex scenarios, especially when handling occlusions and non-rigid deformations.

Z. Zou developed a graph convolutional network (GCN)-based 3D pose estimation model [18] that exploits topological relationships between human joints to enhance 3D body keypoint prediction accuracy. This approach has established new performance benchmarks on both Human3.6M and MPI-INF-3DHP datasets.

K. Ludwig proposed a self-supervised learning method for human pose estimation [19] that demonstrates exceptional performance in low-data regimes while maintaining strong generalization capabilities.

C. Palmero developed a multimodal fusion model [20] that integrates RGB images with depth data to enhance the robustness of body keypoint detection. This approach demonstrates superior performance in challenging illumination and occlusion scenarios, particularly excelling on indoor datasets.

X. Li proposed a temporal convolutional network (TCN)-based video pose estimation model [21] that leverages temporal continuity to optimize body keypoint detection results. The model achieves state-of-the-art performance on the PoseTrack dataset and is particularly suitable for dynamic scene pose estimation.

The aforementioned research demonstrates significant advancements in human body keypoint detection models for sports applications, particularly in terms of accuracy, efficiency, and scenario adaptability. The field has witnessed remarkable progress, from high-resolution networks (HRNet) to Transformer-based PoseFormer architectures, from 2D to 3D pose estimation, and from unimodal to multimodal fusion approaches. These developments have substantially expanded the application of computer vision in sports motion analysis and correction.

Network models for motion detection require lightweight designs, which offer three primary advantages: (1) reduced data communication volume during distributed training, (2) faster cloud-to-edge deployment due to there being fewer parameters, and (3) better suitability for deployment on resource-constrained embedded and mobile devices (e.g., FPGAs). Current lightweight solutions primarily fall into two categories.

The first approach involves architectural simplification by reducing convolutional scale and model parameters. A representative example is Redmon’s YOLOv3-tiny [22], derived from the classic YOLOv3 model [23] through structural pruning. This optimized model achieves a 96.04% reduction in floating-point operations (FLOPs) while significantly lowering deployment costs, enabling widespread practical implementation [24].

The second approach focuses on optimizing standard convolution operations to create lightweight computational units. Howard et al.’s MobileNet [25] decomposes standard convolutions into depthwise and pointwise convolutions, substantially reducing computational parameters. Similarly, Ma et al.’s ShuffleNet [26] employs group convolutions with 1 × 1 kernels to decrease computation load while maintaining information flow through channel shuffling. Han et al.’s GhostNet [27] introduces the ghost module, which replaces portions of standard convolution operations with cost-efficient linear transformations, thereby reducing computational complexity. Compared to architectural simplification, these convolution optimization methods offer greater generality as they can be readily applied to various existing CNN architectures.

Typical lightweight applications of the embedded ghost layer are as follows.

Li et al. proposed an online learning state evaluation method based on a face detection method and head pose estimation [28]. The subnetwork of face detection employs ghost and attention modules. The other, for head pose estimation, applies a Dual-Branch (DB) network. Both of them are lightweight, and the final evaluation model can also be translated to mobile phones.

Liu et al. proposes a YOLO fire detection algorithm based on an attention-enhanced ghost mode, mixed convolutional pyramids, and flame-center detection [29]. The ghost layer reduces redundancy in multi-channel convolutions and significantly lightens the YOLO architecture. The effectiveness of embedding ghost layers was validated across multiple YOLO variants, achieving both reduced false detection rates and improved real-time processing frame rates (FPS).

## 3. Our Method and System

**Figure 2:**
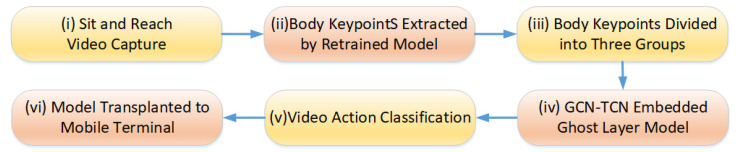
The detection process of the sit-and-reach pose motion in this paper.

As discussed in Section 2, the prevailing motion detection method first extracts human keypoints (a form of dimensionality reduction), then groups them to minimize training data, conducts temporal analysis, and ultimately produces the detection result. This paper first analyzes the characteristics of the sit-and-reach posture detection task. The action detection is divided into two categories; that is, the outputs are ‘correct action’ and ‘wrong action’. The overall scheme flow of this paper has six steps which is shown in Figure 2. Step (i) is to collect the sit-and-reach video. Step (ii) is to extract the body keypoints using a retrained model, which is presented in Section 3.1. Step (iii) is to group multiple keypoints of the human body. Step (vi) is to group the spatio-temporal data of the keypoints of the human body into the Ghost-ST-GCN model proposed. Step (v) is to classify the feature maps output by Ghost-ST-GCN for the sit-and-reach video. Finally, Step (iv) is to migrate to the mobile terminal for deployment.

### 3.1. Self-Train Model of Body Keypoints

**Figure 3:**
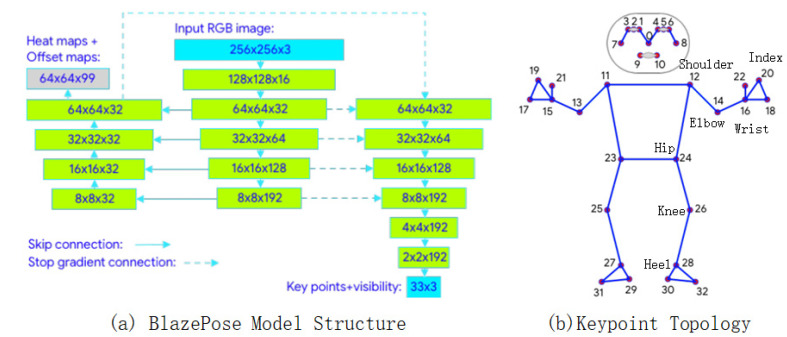
The structure and the proposed body keypoints of the BlazePose model.

The foundation of action detection lies in the extraction of human keypoints, with different actions requiring distinct keypoints. Based on the characteristics of the sit-and-reach test, this paper employs the BlazePose model to extract seven keypoints. The structure of BlazePose is illustrated in Figure 3a. Two publicly available datasets annotated with 33 keypoints (Figure 3b) are referenced.

**Figure 4:**
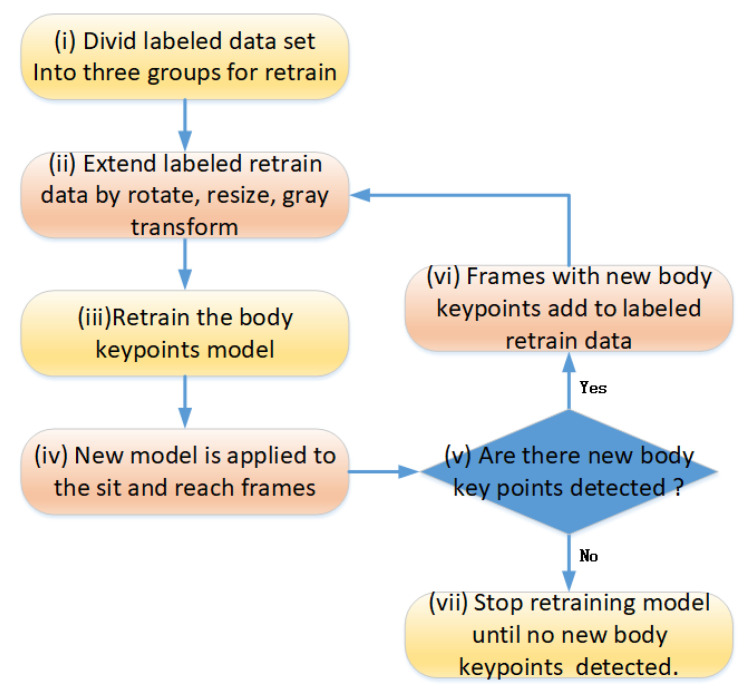
The self-train flow between the labeled data and unlabeled sit-and-reach video frames.

Although there are significant differences in human poses between the labeled datasets (predominantly standing postures with few sitting poses) and the sit-and-reach video frames (exclusively sitting postures), locally scaled and rotated body parts from the former demonstrate high similarity with corresponding regions in the latter. Leveraging this feature, the seven keypoints are divided into three groups for separate retraining of BlazePose model. The grouping strategy is detailed in Section 3.2. During keypoint detection in sit-and-reach video frames, it was observed that while the complete detection of seven keypoints was achieved in some frames, others failed to detect all keypoints. To address this, this paper proposes a self-supervised approach to train all frames, thereby improving the model’s accuracy in detecting seven keypoints, the flowchart for which is shown in Figure 4.

Firstly, the labeled public body keypoints are divided into three groups. Secondly, the group and labeled body keypoints data are extended by image rotation, scale resizing, and grayscale transformation. Thirdly, these extended labeled data are fed to the original body keypoint detection model for retraining. Fourthly, some frames may detect new key points, and other frames may not detect new key points. Fifthly, it is necessary to determine whether there are new body keypoints in the unlabeled frames. If there are, enter the sixth step, select these frames with labeled new body keypoints as new training data, and then return to the third step. Otherwise, if there are no new keypoints in any of the frames, then this round of self-training is introduced. The final keypoint training model will be used in subsequent action detection. The proposed self-train strategy uses the spatial–temporal correlation between sit-and-reach video frames to increase the detection rate of self-trained body keypoint detection.

### 3.2. Body Keypoints Grouping

**Figure 5:**
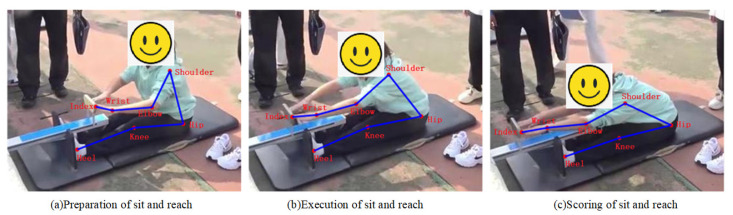
The seven body keypoints extracted from sit-and-reach video frames by the self-train model.

By observing the sit-and-reach video, one can see that this action has very strong symmetry, which can make up for the occlusion of limb joints during video acquisition. For example, in Figure 1, the left side of the juvenile body can be evaluated, but the right side of the body is occluded. After the body keypoint detection is performed, the model can better realize the detection of unilateral keypoints, and the sit-and-reach model only needs to observe the unilateral limb to determine whether the action is correct. Here, only a single keypoint needs to be detected. In order to further simplify the model, this paper proposes detecting seven body keypoints in a set G, which includes the shoulder, elbow, wrist, index, hip, knee, and heel. G={12,14,16,20,24,26,30} or G={11,13,15,19,23,25,29}. In this paper, we no longer distinguish between left and right in the subsequent chapters. These points are taken for self-training, and an example of a sit-and-reach video frame is shown in Figure 5. The red dot indicates the keypoint, and the blue connection indicates the bone connection between the keypoints in Figure 5.

After reducing the number of observation points, the keypoints are grouped. The appropriate grouping can effectively improve the efficiency of the feature network extraction network, and then improve the accuracy of action detection. In this paper, the seven keypoints are divided into three groups, as in Formula (1). The symbol ‘∪’ denotes union operation and k denotes grouping number.

(1) $$G={⋃}_{k=1,2,3}G_{k}$$

The basis for dividing these three groups is to ensure that there are at least two common joints between adjacent groups, as shown in the following Formula (2). The symbol ‘⋂’ denotes the intersection operation, and i and j denote the adjacent set index.

(2) $$num(G_{i}⋂G_{j})\geq 2\;$$

**Figure 6:**
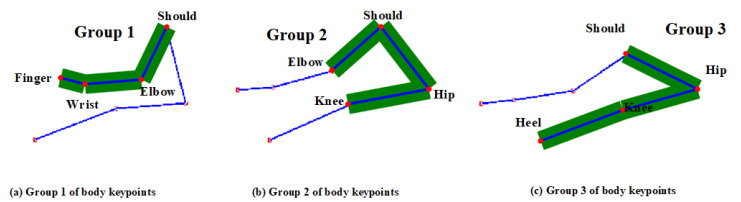
Grouping of body keypoints in the sit-and-reach video frame.

In Formula (2), num( ) represents the number of body keypoints, and the specific grouping is shown in Formula (3) and Figure 6.

(3) $$\left\{\begin{array}{c} G_{1}=\{12,14,16,20\}\\ G_{2}=\{12,24,26,30\}\\ G_{3}=\{12,14,24,26\} \end{array}\right.$$

During the sit-and-reach test, subjects frequently exhibit several incorrect postures, such as “knees remain unbent,” “inadequate forward bending at the waist,” and “elbows remain unbent.” These errors may occur individually or in combination within a single subject’s performance. To streamline the modeling process, this paper categorizes all such instances as incorrect movements. The multiple keypoints corresponding to these errors serve as the basis for seven groupings.

**Figure 7:**
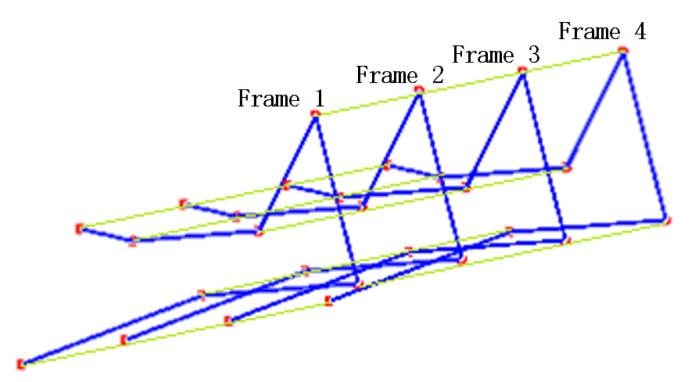
Spatio-temporal connection of keypoints in 4 frames.

The seven keypoints identified in each frame of the video can be used for feature extraction via a graph convolutional network (GCN), represented by the blue connecting lines between keypoints in a single frame, as shown in Figure 7. Concurrently, variations in corresponding keypoints across consecutive frames capture temporal features, which can be extracted using a temporal convolutional network (TCN). This is illustrated by the light green connecting lines spanning four consecutive frames in Figure 7. Multiple keypoints of the human body form a graph structure. The keypoints are the nodes of the graph, the line between the keypoints is the edge of the graph, and the node information refers to the coordinates and confidence.

### 3.3. Proposed Ghost-ST-GCN

**Figure 8:**
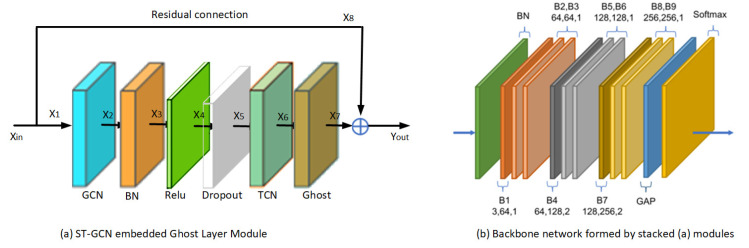
Proposed ST-GCN embedded ghost module and stacked module to backbone network.

In this paper, an ST-GCN with a ghost layer is proposed. One basic module is the combination of one spatial GCN, Batch Normalization (BN), Relu activate (Relu), Dropout, TCN, and ghost layers, which is shown in Figure 8a. To stabilize the training, a residual connection is added for each module. One BN layer and Global Average Pooling (GAP) + Softmax layer are added at the beginning and end of the nine basic modules to obtain the proposed network of the sit-and-reach detection model in this paper, which is denoted as B1~B9 and shown in Figure 8b. The three numbers of each block Bi represent the number of input channels, the number of output channels, and the stride step. For example, B4 is 64,128,2; that is, the number of input channels of the module is 64, the number of output channels is 128, and the stride step is 2. To prevent the issue of vanishing/exploding gradients when stacking multiple layers of modules, this paper incorporates a residual connection [30] into each fundamental module B. When the number of input channels differs from that of output channels, a 1 × 1 convolution is inserted along the residual path to align the channel dimensions with the output.

The core formula of the graph convolution network (GCN) is as in Formula (4)

(4) $$H^{\left(l+1\right)}=\sigma ({\tilde{D}}^{-\frac{1}{2}}\tilde{A}{\tilde{D}}^{-\frac{1}{2}}H^{\left(l\right)}W^{\left(l\right)})$$

where $H^{\left(l\right)}$ is the node feature matrix of the (l) layer (initial $H^{\left(0\right)}=X$, X is the original input feature matrix); $\tilde{A}$ is the adjacency matrix with self-ring, and the calculation formula is Formula (5)

(5) $$\tilde{A}=A+I$$

where I is the identity matrix and A is the adjacency matrix. $\tilde{D}$ is the degree matrix of $\tilde{A}$. $\tilde{D}$ represents the number of neighbors of each node, and is a diagonal matrix, as shown in Formula (6).

(6) $$\tilde{D}\left[i\right]\left[i\right]=\sum_{j}\tilde{A}\left[i\right]\left[j\right]$$

$W^{\left(l\right)}\;$ is the learnable weight matrix of the (l) layer; σ(·) is the activation function (e.g., ReLU); the GCN layer can expand the channel dimension using multiple graph convolution kernels. Other layers, such as Batch Normalization (BN) and Dropout, do not alter the channel dimension. In contrast, the TCN layer performs filtering along the temporal axis while preserving the original channel dimension. For instance, in Block B1, the input features have a channel dimension of 3, while the output dimension is 64.

As illustrated in Figure 8a, the input of module B1 is denoted as Xin, with dimensions Cin, T, and N, while its output is Yout (Cout, T, V), where C represents the channel count, representing the keypoint coordinates and its confidence. T denotes frame number, and V indicates the number of keypoints. The GCN layer can expand the output channel dimension by employing multiple convolutional kernels for spatial graph convolution. Within B1, Cin = 3 and Cout = 64. The channel count of the GCN layer output X2 is designated as 32, half of the Cout. The output channel count of subsequent layers—BN, ReLu, Dropout, and TCN—preserve the channel count and it remains unchanged. Within the ghost layer, its input X_6_ is from the TCN and undergoes a simple linear transformation to become X_6_′. Subsequently, X_6_ and X_6_′ are processed through channel concatenation, yielding X_7_. Thus, the channel count of the ghost layer output X_7_ is 64.

**Figure 9:**
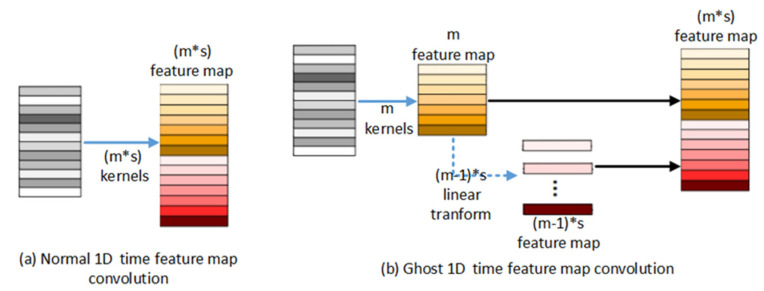
One-dimensional temporal series convolution embedded ghost layer schematic diagram.

The steps for embedding a ghost layer into the ST-GCN module are shown in Figure 9b, which shows a one-dimensional temporal series convolution. In Figure 9a, assuming that the original temporal series is m∗s after m∗s one-dimensional convolution kernel operations to obtain m∗s feature maps, then after embedding the ghost layer in Figure 9b, only m one-dimensional convolution kernels are used to obtain m feature maps, and then the m feature maps are transformed into $\left(m-1\right)*s$ feature maps by $\left(m-1\right)*s$ linear transformations (this transformation can be replaced by a 1 * 1 convolution). Together with the original m feature maps to form m∗s feature maps, the number of one-dimensional filter cores is reduced and the amount of calculation without changing the input and output is reduced.

**Figure 10:**
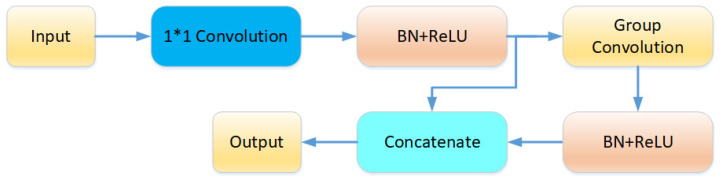
The structure of the proposed embedding ghost layer.

For embedding the ghost module, this paper designs the ghost layer as in Figure 10. For a specific input X with a size of (H,W,M), $X_{1}$ is first generated by a 1∗1 convolution with a size of (H,W,M), that is, the intrinsic feature map mentioned above. M is the number of channels in the feature map, and the value in the ghost structure of this paper is half of the number of output channels. After passing through BN(Batch Normalization) and the activation function, the network takes $X_{1}$ as the input and uses group convolution to generate N redundant feature maps $X_{2}$, where $N+M=C_{out}$, where $C_{out}$ is the number of output channels. Finally, $X_{1}$ and $X_{2}$ are spliced to generate the final output temporal feature map.

The embedding of the ghost module relies on two basic operations.

(i) Real Convolution: The original feature map is generated by a standard convolution operation. Usually, this is an operation with a large amount of calculation, using a traditional convolution kernel. For the input X and the convolution kernel W, the real convolution can be expressed as Formula (7):

(7) $$X_{real}=W_{real}*X$$

(ii) Ghost Convolution: A pseudo-feature map is generated by a small 1∗1 convolution kernel $W_{ghost}$. This operation has a small amount of calculation, but can generate additional feature maps. Pseudo-convolution can be expressed as Formula (8)

(8) $$X_{ghost}=W_{ghost}*X_{real}$$

Finally, the real feature map and the pseudo feature map are spliced to obtain the output of the ghost module, where concat() means feature map channel concatenation:

(9) $$Y=concat(X_{real},X_{ghost})$$

Through these, the ghost layer can generate a large number of feature maps with less computation, thus significantly reducing the computational overhead.

## 4. Experiment and Discussion

### 4.1. Dataset Preparation

**Figure 11:**
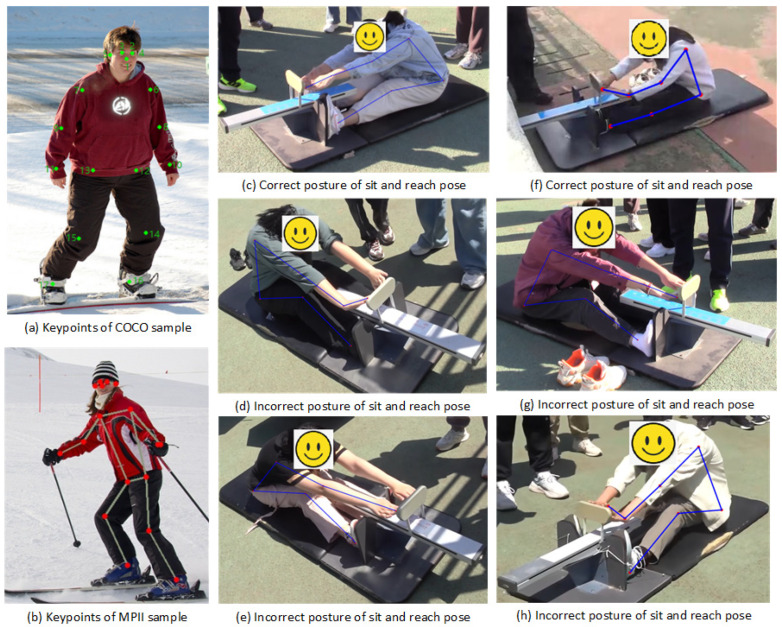
Samples of the COCO and MPII datasets and the sit-and-reach pose.

The training data for human keypoints annotation in this study were sourced from publicly available datasets: COCO (keypoint track) [31] and MPII (Human Pose Dataset) [32]. Representative samples of labeled keypoints are illustrated in Figure 11a,b. Figure 11c demonstrates the correct sit-and-reach pose, while Figure 11f shows the correct intermediate pose. Although the wrist is not parallel to the arm in this case, the fingertips have not yet passed beyond the toe. In contrast, Figure 11d,e,g,h illustrate an incorrect pose where the pose distance scores are deemed invalid. This judgment is based on the criteria that after the fingertip passes the toe, the thigh and calf must maintain full extension while simultaneously requiring the wrist to remain parallel to the arm.

The sit-and-reach videos were obtained from a video-sharing platform, featuring physical fitness assessment videos of first- and second-year university students. Prior to training, all video data underwent de-identification processing to ensure privacy protection. The dataset comprises 1200 video clips of sit-and-reach tests, with each clip containing one participant. Demographic distribution includes 586 males and 614 females, aged between 16 and 22 years. Manual annotation categorized each video into the correct pose, with 1014 clips, and incorrect poses, with 186 clips. The video resolution is 640 × 360 pixels, with each clip capturing a single individual performing the seated forward bend test. Videos were recorded at angles ranging from 15 to 55 degrees below horizontal, lasting 30–60 s at 25 FPS (yielding 750~1500 frames). These frames were partitioned into 60 equal segments. From each segment, the frame with the highest aggregate confidence score across all seven keypoints was selected. If any keypoint was undetected in the chosen frame, its coordinates were supplemented via linear interpolation between the nearest adjacent frames, where the missing keypoint was successfully identified.

### 4.2. Self-Train

The existing datasets annotated with COCO and MPII formats lack labels for the fingertips. To address this, the BlazePose model is first utilized to perform incremental annotation by detecting the fingertips. Subsequently, the seven keypoints are divided into three distinct groups for transfer learning using the BlazePose model: (1) Upper Limb, which includes the index (finger), wrist, elbow, and shoulder keypoints; (2) Trunk, which includes the shoulder and hip keypoints; and (3) Lower Limb, which includes the hip, knee, and heel keypoints.

Prior to training, the annotated dataset is augmented through rotation and scaling transformations. This augmentation aims to align the postures of the keypoints within each group more closely, with the typical postures observed during the sit and reach test. This process yields three separate retrained keypoint detection models, each specialized for its respective group. These three models are then applied to detect the seven keypoints in videos of the sit-and-reach test. The BlazePose model is retrained, and the labeled dataset is rotated and scaled before training.

The seven keypoint detection results of the proposed self-train BlazePose model for the sit-and-reach video are shown in Table 1, in which OKS (Object Keypoint Similarity [31]) is used to measure the accuracy of body keypoint detection. OKS measures the agreement between predicted and ground-truth keypoints, normalized by object scale and keypoint visibility. The larger the value, the worse the labeling effect on this point across the entire dataset; the smaller the value, the better the labeling effect of this point across the whole dataset.

A comparison of the keypoint detection OKS for HRnet-w48 and BlazePoze Lite before and after self-training is presented in Table 1. The results indicate that the keypoint detection rates of the two models are comparable. However, HRnet-w48 has a significantly higher computational cost, approximately 35 GFLOPs, whereas BlazePose Lite requires only 2.7 MFLOPs. Consequently, BlazePoze is clearly more suitable for practical applications in computing resource-constrained mobile scenarios. Following self-training, the model exhibited a significant improvement in the keypoint detection rate of approximately 9%~11%. This result clearly demonstrates the efficacy of the self-train method proposed herein.

### 4.3. Ablation Experiment

The model is tested using the sit-and-reach video and optimized from the model with 9 modules stacked, in which GCN+TCN+Ghost and residual connection (full connection layer) is used, and the specific detection performance and computational cost optimization process are shown in Table 2.

As evidenced in Table 2, when stacking GCN-TCN-Ghost modules 3 to 15 times, the accuracy initially increases before exhibiting a slight decline, peaking at the nine-layer configuration. Models with residual connections achieve significantly higher accuracy. Although embedding ghost layers results in a marginal accuracy reduction, they substantially reduce model parameters to 2.2 M—less than half the 3.1 M parameters of the 9-stacked modules without a ghost layer—making the architecture better suited for mobile deployment. Consequently, this study adopts the nine-layer stacked GCN-TCN-Ghost model as the final architecture.

### 4.4. Lightweight Estimation of Ghost Layer

The model streamlining in this paper is achieved by embedding ghost layers into the GCN-TCN modules, as illustrated in Figure 8a. The primary computational load within this module resides in the GCN and TCN components. When stacking the nine modules, they exhibit varying input/output channel configurations (Figure 8b), and the GCN modules employ different convolution strides. Modules with larger strides incur relatively lower computational costs. The FLOPs (floating-point operations) of individual modules also differ slightly. Body keypoints are divided into three groups, each containing four nodes connected linearly (1-2-3-4). The TCN maintains constant channel dimensions, with a temporal filter length of nine frames. In the ghost layer, the number of expanded channels equals half of the output channels. If the input channel count equals the output channel count, half of the input channels are randomly discarded at the input side, thereby halving the channel dimension prior to processing. The FLOPs for modules B1–B9 are detailed in Table 3. As shown in the table, embedding ghost layers reduces computational complexity to approximately 70% of the original complexity. The lightweight estimation is defined as follows:

(10) $$L_{ratio}=\frac{{FLOPs}_{No\;Ghost}-{FLOPs}_{Ghost}}{{FLOPs}_{No\;Ghost}}\times 100\%$$

### 4.5. Sit and Reach Detection

The input dimension of the proposed Ghost-ST-GCN is $\left(N,C,T,V,M\right),$ where N represents the number of videos, and there are usually eight videos in a batch. C represents the characteristics of the joint; usually a joint contains x,y and acc, where x,y are the coordinates of the keypoint, and acc is the confidence. T represents the number of frames, which is 60; V represents the number of body keypoints, which are grouped according to formula 3, and the value is 4; M represents the number of people in the video, which is one.

**Figure 12:**
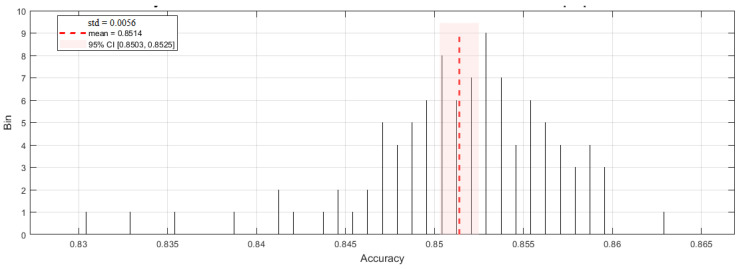
Accuracy histogram statistics from 20 random shuffles with 5-fold cross-validation of the proposed model.

The videos were partitioned into training and testing sets in an 8:2 ratio. This resulted in 811 correct pose samples for training (203 for testing) and 149 incorrect pose samples for training (37 for testing). To evaluate model performance, stratified 5-fold cross-validation (K = 5) was implemented 20 times with a random shuffle. Due to the limited number of test samples, affine transformation was employed here to augment the test dataset. This transformation can simulate variations in shooting angle and scale, making it highly suitable for altering the coordinates of keypoints. The keypoint confidence score is added using a random value, which follows a Gaussian distribution with a mean of 0 and a variance of 0.05. Figure 12 shows a histogram of the model detection accuracy statistics after 20 random shuffles with 5-fold cross-validation. The model achieved a mean accuracy of 0.8514 (std = 0.0056) over 100 tests, in which minimum and maximum values of 0.8308 and 0.8633, respectively, were achieved. The 95% confidence interval was [0.8503, 0.8525].

In this study, the detection accuracy of four methods (TS-LSTM [33], TimeSFormer [34], ST-GCN, Ghost-ST-GCN) on the sit-and-reach test is shown in Table 4. The proposed Ghost ST-GCN achieves higher detection accuracy compared to the baseline ST-GCN, with further improvements observed after self-training on body keypoints extracted from sit-and-reach video frames. Additionally, the computational efficiency of the proposed model is demonstrated through a significant reduction in both the number of trainable parameters and the model size. These results indicate that Ghost ST-GCN effectively lightens the model architecture while maintaining high detection accuracy.

As shown in Table 4, the proposed Ghost-ST-GCN model achieves comparatively higher accuracy than the classical ST-GCN approach while maintaining a more compact architecture. Note that this model operates collaboratively with the BlazePose Lite module, which itself has a minimal parameter size of ~1.2 M. Three keypoint detection models require retraining in this framework (totaling ~3.6 M), resulting in a combined parameter count of ~4.8 M for the full pipeline. This represents a substantial advantage over TS-LSTM and TimeSFormer models, which typically require hundreds of megabytes of parameters.

Furthermore, Table 1 and Table 3 collectively demonstrate that the self-train strategy employed to enhance keypoint detection rates directly contributes to improved accuracy in the seated forward bend action recognition task.

### 4.6. Analysis of Experimental Results

The experimental results demonstrate the following key findings: As shown in Table 1, the self-training strategy for keypoints significantly improved the detection accuracy. Table 2 presents an ablation study that identifies the optimal number of stacked modules. Table 3 provides a quantitative analysis of the computational load after integrating the ghost module. Table 4 indicates that the proposed method exhibits substantial advantages in lightweight design compared to three other action detection approaches, with its accuracy already meeting practical application requirements.

Furthermore, the computational complexity (in FLOPs) can be utilized to estimate the inference time on mobile platforms. The total computational cost of the three BlazePose Lite models combined with the nine-layer Ghost-GCN-TCN architecture is approximately 500 MFLOPs. On the Raspberry Pi 4 platform, whose GPU delivers a theoretical peak performance of roughly 24 GFLOPs, and assuming a practical utilization efficiency of only 20%, the estimated inference time for the complete model is approximately 100 ms. In contrast, on the Apple iPhone 14 platform equipped with the A15 Bionic chip and its 16-core Neural Engine (theoretical performance: ~15.8 TOPS or 15,800 GFLOPs/s), and assuming a 50% utilization efficiency, the estimated inference time falls below 1 ms. This demonstrates the model’s capability for real-time processing and instantaneous response during live video capture.

## 5. Conclusions

The sit-and-reach exercise is a relatively simple movement that involves fewer keypoints on the body, with joint connections forming simple linear chains. The range of correct and incorrect postures is limited. The proposed self-training keypoint extraction method, fixed keypoint grouping strategy, and lightweight Ghost-STGCN model in this paper can effectively detect the sit-and-reach exercise. However, for multi-players or sheltered pose detection, such as in gymnastics or yoga, more accurate keypoint extraction models, time-varying keypoint grouping strategies, deeper spatial graph features, and enhanced temporal joint variation characteristics are required. This constitutes the direction for future improvements to extend the proposed method to complex movement detection. Furthermore, integrating Transformer structures, which are more suitable for seq-to-seq modeling, could also be explored for complex movement detection.

In the present study, the integration of ghost layers into the stacked GCN-TCN modules reduces the graph convolutional kernels, leading to a lightweight overall model. However, the computational cost of STGCN remains dependent on the number of nodes. For complex action detection tasks involving a large number of keypoints, the proposed method requires further improvement. Future work will incorporate lightweight approaches such as SGCN (Simplifying Graph Convolutional Network) [35] and DGNN (dynamic graph neural network) [36] to streamline the STGCN model, enabling real-time detection on mobile devices. This advancement will facilitate exercise guidance for a broader population during physical activities.

## Figures and Tables

**Table 1:** OKS testing of seven keypoints before self-training in unlabeled videos.

		OKS Before Self-Train	OKS After Self-Train
HRnet-w48	Train set	87.56%	**96.35%**
	Test set	77.68%	**86.71%**
BlazePose	Train set	82.73%	**93.61%**
	Test set	75.18%	**85.24%**

**Table 2:** The accuracy result of the ablation experiment using the proposed model.

Number of Modules Stacked	No Residual No Ghost	No Residual Ghost	Residual No Ghost	Residual Ghost
3	57.17%	55.92%	61.67%	60.25%
6	64.75%	62.92%	68.08%	67.00%
9	73.17%	72.08%	85.80%	**85.20%**
12	72.25%	71.17%	83.33%	82.33%
15	70.92%	69.50%	82.33%	79.58%

**Table 3:** The FLOP of the stacked GCN-TCN modules with or without a ghost layer.

Module Number	Cin→Cout	Stride Step	No-Ghost FLOPs	With-Ghost FLOPs	Lightweight (%)
1	3→64	1	17,909,760	5,099,520	71.48%
2	64→64	1	19,783,680	6,036,480	69.47%
3	64→64	1	19,783,630	6,036,480	69.47%
4	64→128	2	74,895,360	21,841,920	70.87%
5	128→128	1	78,827,520	23,810,000	69.72%
6	128→128	1	78,827,520	23,810,000	69.72%
7	128→256	2	299,151,360	86,875,200	70.96%
8	256→256	1	314,880,000	94,740,480	69.93%
9	256→256	1	314,880,000	94,740,480	69.93%

**Table 4:** Detection accuracy of several models on sit-and-reach videos.

Detect Accuracy	Before Self-Train	After Self-Train	Model Parameter	Model Disk Size
TS-LSTM	70.08%	82.25%	98 M	382 M
TimeSFormer	73.17%	83.33%	121.4 M	485 M
ST-GCN	76.26%	84.74%	2.5 M	11 M
Ghost-ST-GCN	**76.43%**	**85.20%**	**1.2 M**	**5 M**

## Data Availability

Due to privacy and copyright concerns, the datasets generated and/or analyzed during the current study are not publicly available but can be obtained from the corresponding author upon reasonable request.
